# Ser/Thr Kinase-Like Protein of *Nicotiana benthamiana* Is Involved in the Cell-to-Cell Movement of *Bamboo mosaic virus*

**DOI:** 10.1371/journal.pone.0062907

**Published:** 2013-04-30

**Authors:** Shun-Fang Cheng, Meng-Shan Tsai, Chia-Lin Huang, Ying-Ping Huang, I-Hsuan Chen, Na-Sheng Lin, Yau-Heiu Hsu, Ching-Hsiu Tsai, Chi-Ping Cheng

**Affiliations:** 1 Department of Life Sciences, Tzu Chi University, Hualien, Taiwan; 2 Graduate Institute of Biotechnology, National Chung Hsing University, Taichung, Taiwan; 3 Institute of Plant and Microbial Biology, Academia Sinica, Nankang Taipei, Taiwan; Nanjing Agricultural University, China

## Abstract

To investigate the plant genes affected by *Bamboo mosaic virus* (BaMV) infection, we applied a cDNA-amplified fragment length polymorphism technique to screen genes with differential expression. A serine/threonine kinase-like (NbSTKL) gene of *Nicotiana benthamiana* is upregulated after BaMV infection. NbSTKL contains the homologous domain of Ser/Thr kinase. Knocking down the expression of *NbSTKL* by virus-induced gene silencing reduced the accumulation of BaMV in the inoculated leaves but not in the protoplasts. The spread of GFP-expressing BaMV in the inoculated leaves is also impeded by a reduced expression of *NbSTKL.* These data imply that NbSTKL facilitates the cell-to-cell movement of BaMV. The subcellular localization of NbSTKL is mainly on the cell membrane, which has been confirmed by mutagenesis and fractionation experiments. Combined with the results showing that active site mutation of NbSTKL does not change its subcellular localization but significantly affects BaMV accumulation, we conclude that NbSTKL may regulate BaMV movement on the cell membrane by its kinase-like activity. Moreover, the transient expression of *NbSTKL* does not significantly affect the accumulation of *Cucumber mosaic virus* (CMV) and *Potato virus X* (PVX); thus, NbSTKL might be a specific protein facilitating BaMV movement.

## Introduction

Protein phosphorylation is a reversible post-translational modification that regulates the cell functions in response to various environmental stimulations. However, pathogens often take advantage of the cell’s existing systems to complete their life cycles. An increasing number of studies has shown that signaling through phosphorylation of cellular and viral proteins play key roles during pathogen entry, intracellular movement, and exit from host cells [1].

In studies regarding plant viruses, phosphorylation of viral-encoded proteins has been shown to modulate symptom expression or pathogenicity [2]–[5], influence the protein-protein interaction [6], and inhibit replication protein binding to the viral RNA of *Cucumber necrosis virus*, and then further affect viral replication [7], [8]. Moreover, phosphorylation of viral movement proteins (MP) also regulates the cell-to-cell movement of plant viruses [9]–[11]. *Tobacco mosaic virus* (TMV) is one of the most intensive study models in the field of cell-to-cell movement of plant viruses [12]. Previous studies have shown that the TMV MP is phosphorylated at its C-terminus by a plasmodesmal-associated protein kinase [13], [14]. In a further investigation on different phosphorylation sites of TMV MP, Trutnyeva *et al*. demonstrated that sequential phosphorylation of MP may regulate the function of MP in cell-to-cell transport [15]. Phosphorylation of TMV MP is also important in subcellular localization of MP because mutation on the phosphorylation sites reduces the association of MP with ER, plasmodesmata, and microtubules; these associations are linked to the virus replication and delivery of the vRNA complex to the neighboring cells through plasmodesmata [11], [16]–[18]. Ouko *et al*. recently demonstrated that targeting of TMV MP to the plasmodesmata is reduced in microtubule growth-deficient tobacco mutants, and consequently, the mutants are more resistant to TMV infection [19]. Furthermore, TMV MP can be phosphorylated at Thr^104^ by an ER-associated kinase of *N. tabacum*; mutant that mimicking the phosphorylation results in a severe defect on cell-to-cell movement [20]. In a recent study, TGBp1, one of the MP of *Potato virus X* (PVX), was also found to be phosphorylated possibly by casein kinase 2 (CK2)-like activity from an *N. tabacum* extract [21]. However, the physiological function of this phosphorylation has not been determined.

*Bamboo mosaic virus* (BaMV) infects over 13 species of bamboo and lowers the quality of bamboo shoots significantly, resulting in high economic losses in Taiwan [22]. BaMV is a single-stranded positive-sense RNA virus belonging to the genus *Potexvirus*. BaMV usually causes mosaic symptoms on bamboo leaves and on *N. benthamiana* [23]. The genome of BaMV is approximately 6.4 kb in length and encodes 5 open reading frames (ORFs). ORF1 protein contains 3 domains that exhibit guanylyltransferase [24], helicase [24], and RNA-dependent RNA polymerase activities [25] from the N- to C-terminus, respectively. ORFs 2–4 of BaMV organizing in an overlapping triple gene block (TGB) have the features of MP required for virus cell-to-cell movement [26], [27]. The TGBp1 (P28) of BaMV is found mainly in the cell wall fraction, if at all; TGBp1 found in the cytoplasm and nuclei is associated with electron-dense crystalline bodies (EDCBs) [28]. The last ORF is translated into a coat protein, which has multiple functions [23], [29], [30].

To understand the interactions between host plants and BaMV, we adopted an approach of cDNA-amplified fragment length polymorphism (AFLP) to identify several host proteins involved in BaMV infection [31]. One protein, which shares a conserved domain as a protein kinase, was upregulated after BaMV inoculation. We further analyzed how this gene product is involved in BaMV infection in this study.

## Materials and Methods

### Total RNA Extraction

Total RNA was extracted from *N. benthamiana* plants using hot phenol and LiCl precipitation as described previously [31].

### Full-length cDNA Cloning of NbSTKL

Rapid amplification of the 5′- and 3′-ends of NbSTKL was performed by SMART RACE cDNA amplification kit (Clontech Laboratories, Inc. Mountain View, CA, USA ) followed the manufacturer’s instructions. Briefly, the 3′RACE was used to obtain the very 3′-end sequences of NbSTKL by using the 3′RACE CDS primer A for RT followed by PCR with Universal Primer A Mix (UPM) and T-CA/M-AC#5F primer to obtain nt 796–1101. Nucleotides 603 to 969 of NbSTKL were obtained by 5′RACE with NbSTKL elongation reverse primer and UPM. Nucleotides 1 to 803 of NbSTKL were amplified by PCR using NbSTKL specific primer STKF and sequence of the primer from a *N. benthamiana* cDNA sequence in gene bank (EX534117). The resulting product were cloned into pGEM-T Easy (Promega, Madison, WI, USA) vector and sequenced.

### Constructs

*Tobacco rattle virus* (TRV)-based VIGS system was used to knock down the expression of host genes. Plasmids pTRV1, pTRV2 and pTRV2 with PDS (phytoene desaturase) gene were kindly provided by Dr. Baulcombe (Department of Plant Sciences, University of Cambridge, UK). The cDNA fragment derived from cDNA-AFLP was released from pGEM-T Easy vector by *EcoR*I digestion and cloned into TRV2 plasmid. The resulting plasmid was then transformed into *Agrobacterium tumefaciens* C58C1 strain for knocking down the expression of endogenous NbSTKL gene in *N. benthamiana* as described previously [31]. For transient expression of NbSTKL, the ORF is amplified by primers STK XbaI/F and STK del stop SalI/R and cloned into pBIN-mGFP vector by restriction enzyme sites *Xba*I and *Sal*I to generate pBIN-NbSTKL-GFP. To create the NbSTKL active site mutant, NbSTKL/D224A, primers STK D to A/F and STK808–829/R are used to generate mega primer which changes a.a. 224 aspartic acid to alanine. The mega primer is then used for second PCR with STK504-528/R primer. The resulting PCR product is cloned into *Hind*III and *Nde*I restriction enzyme site of pBIN-NbSTKL-GFP. See Table 1 for all the primers used in this study.

**Table 1:** Primers used in this study.

Name of the primer	Sequence of the primer
NbSTKL elongationreverse primer	5′-CTGAACCTTCCTCAATACAGAAGCAA-3′
NbSTKL elongation forward primer	5′-TCGTTTGTCTGACGTGGTGACT-3′
STKF	5′-GAAGCTTGGAAAACCATCT-3′
T-CA/M-AC#5F	5′-CAAGAAGTCGTCAGTCACC-3′
NbSTKL/F	5′-ATGGGGAATTGTT TTGGAGCT-3′
NbSTKL/R	5′-GTTGAACTCTGAGAGCACTC-3′
ACF	5′-GATGAAGATACTCACAGAAAGA-3′
ACR	5′-GTGGTTTCATGAATGCCAGCA-3′
STK XbaI/F	5′-GTCTAGAATGGGGAATTGTTTTGGAGCT-3′
STK del stop SalI/R	5′-GGTCGACGCCACGGCCGTTTGTCTG-3′
STK504-528/F	5′-GTTACTTGTTTATGAATTTATGCAG-3′
STK808-829/R	5′-GGCCTGGTTCAACATATTCTGG-3′
STK D to A/F	5′-TACAGAGCGTTCAAGGCCTCCAAC-3′

### NbSTKL Knockdown and Virus Inoculation

*N. benthamiana* plants were grown in pots at 28°C in a growth chamber under 16 h light/8 h dark cycle for about one month before *Agrobacterium*-infiltration. *A. tumefaciens* containing plasmid pTRV1 or pTRV2-derivatives was grown to OD_600_ = 1 and mixed in 1∶1 ratio followed by infiltration onto three leaves above the cotyledons of each plant. Ten days after infiltration, BaMV viral particle (0.5 μg) was mechanically inoculated onto the fourth and fifth leaves above the infiltrated leaves [31].

### Protoplast Inoculation

Protoplasts were isolated from *NbSTKL*-knockdown *N. benthamiana* 10 days post *Agrobacterium*-infiltration. Approximately 2 g of *N. benthamiana* leaves were harvested from the fourth leaf above those infiltrated leaves and subjected for protoplast isolation. Each protoplast sample approximately 5×10^5^ cells was inoculated with 1μg of BaMV viral RNA by PEG method and incubated for 24 h at 25°C under a constant light as described previously [32].

### Semi-quantitative RT-PCR

To confirm the expression profile of NbSTKL after BaMV inoculation or after gene knockdown, the first strand cDNA was synthesized from 1 mg total RNA of control or gene-knockdown plants with d(T)_39_ primer. To confirm the cDNA-AFLP result, NbSTKL con/F and NbSTKL con/R primers were used for PCR reaction. Endogenous actin was amplified as control for equal cDNA amount in each reaction by primers ACF and ACR.

### Western Blot Analysis

The total proteins were extracted from the virus inoculated leaves at 5, 7 days post inoculation and detected with rabbit anti-viral (BaMV, PVX, and CMV) coat protein antibodies. The relative levels of the Coomassie blue-stained Rubisco large subunit (rbcL) in gels were determined and used for the normalization of the coat protein signals [31].

### Measurement of GFP Foci

About 10 µg pCBG plasmid [26] was inoculated onto the fourth leaf above the infiltrated leaf of control (Luciferase-knockdown) or NbSTKL-knockdown *N. benthamiana*. At 6 dpi, the green fluorescent foci in the inoculated leaves were captured with an Olympus IX71 inverted microscope using a filter set of an excitation filter (460 to 495 nm), an emission filter (510 nm), and a dichromatic mirror (505 nm). The green fluorescent area of microscopy images was counted by Image J software.

### Separation of Cytoplasm and Membrane Fractions

About 1 g transiently expressed-leaves were ground with liquid nitrogen and 2 ml pre-chilled buffer A (10 mM sodium phosphate pH 7.4, 100 mM NaCl, addition of 2 mM β-mercaptoethanol and protease inhibitor just before use) was added and followed by a 12000 g centrifugation for 5 min at 4°C. After centrifugation, the supernatant which represented the cytoplasm was transferred to a new 1.5 ml microtube and kept at −20°C. The pellet containing the membrane fraction was washed twice with 2 ml buffer A in a 12000 g centrifugation for 5 min each time at 4°C. The pellet was then dissolved in 2 ml buffer A with 0.5% SDS and stirred for 30 mins at 4°C. The supernatant containing the membrane proteins was collected at 12000 g centrifugation for 5 mins at 4°C.

### Kinase Activity Assay

The soluble fractions of NbSTKL-GFP, NbSTKL/D224A-GFP, NbSTKL/G2A-GFP, and GFP from transiently expressed-leaves were purified by Magnetic GFP-Trap (Chromotek, Germany). The bound samples with beads were resuspended in 20 µl reaction buffer (20 mM Tris-HCl pH 7.5, 10 mM MgCl_2_) containing 80 nM [γ-^32^P]ATP with specific activity 6000 Ci/mmol and incubated at 25°C for 20 mins. The reactions were stopped by adding the sample buffer (5.125 mM Tris-HCl pH 6.8, 10% SDS, 10% glycerol, 0.005% bromophenol blue, and 2.5% β-mercaptoethanol) and boiled for 5 mins. The samples were separated by 10% SDS-polyacrylamide gel, and exposed for 12 hours. The radioactive signals were scanned with BAS-2500 phosphoimager (FUJIFILM, Japan).

## Results

### Expression of Ser/Thr Kinase-like Gene is Upregulated After BaMV Infection

**Figure 1:**
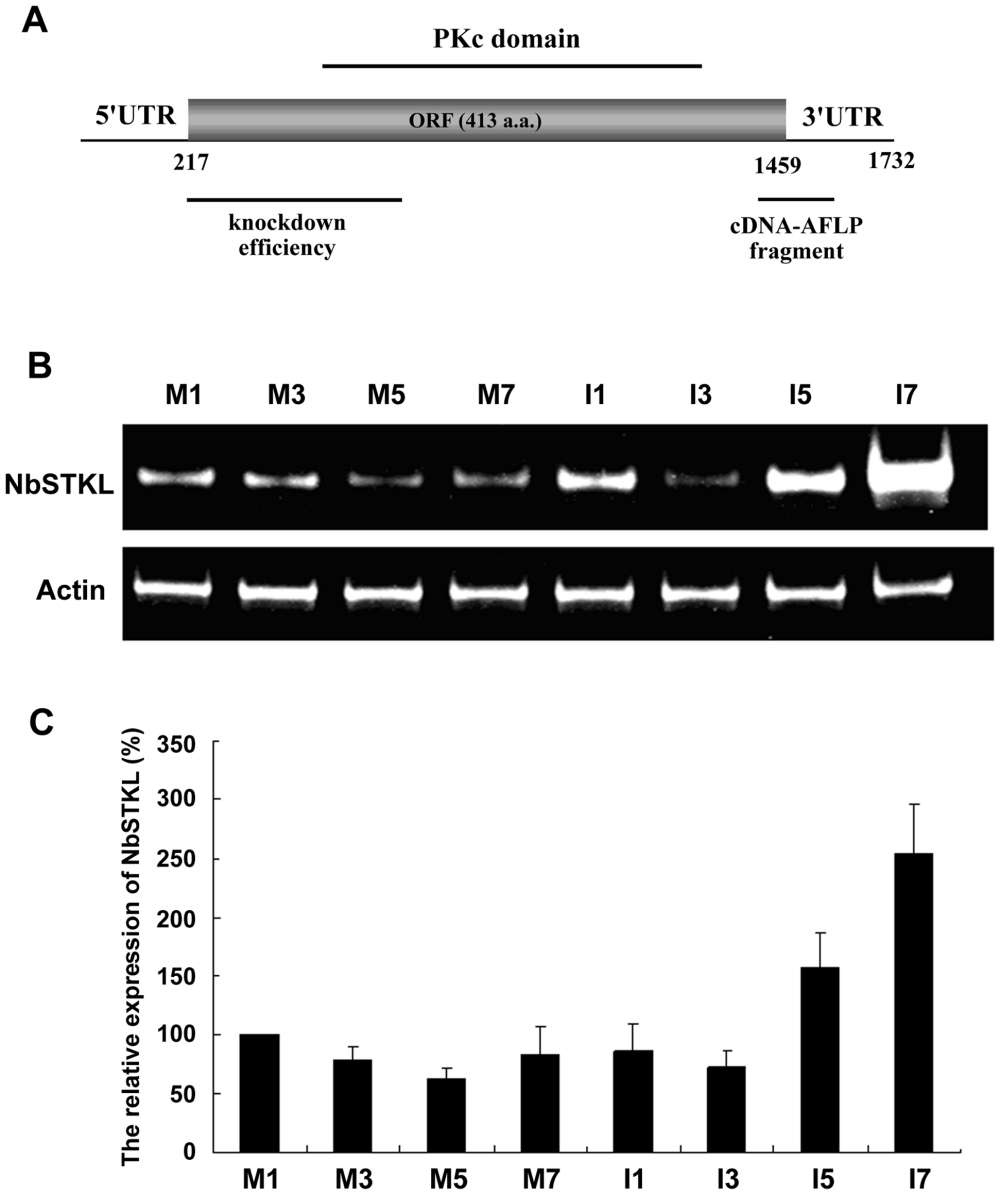
The cDNA feature and the expression of NbSTKL after BaMV inoculation. **A.** The full-length cDNA of NbSTKL is predicted to contain a 413-amino acid open reading frame. The 5′ and 3′ UTRs are indicated. The cDNA-AFLP fragment indicated was used for knockdown experiment and the fragment denoted as knockdown efficiency was used for measuring the expression of NbSTKL. The catalytic domain of protein kinase (PKc) is shown above the ORF. **B.** Semi-quantitative RT-PCR was used to detect the expression of NbSTKL (top panel) and actin (down panel) as an internal control. The RNA was extracted from BaMV inoculated (I) or mock inoculated (M) at 1, 3, 5, 7 day post inoculation of *N. benthamiana* indicated. **C.** The expression level of NbSTKL in mock inoculation at 1dpi was set as 100%. The results represent the average from three independent experiments.

A cDNA-AFLP assay was performed to study the differential gene expression in *N. benthamiana* plants after BaMV inoculation [31]. One identified gene upregulated at 5 and 7 d post-inoculation (dpi) contains a serine/threonine (Ser/Thr) kinase-like domain (denoted as NbSTKL) after blasting the sequence to the available database. To confirm that this gene has been regulated after BaMV infection, we inoculated *N. benthamiana* with BaMV and performed a semi-quantitative RT-PCR to demonstrate that the expression of this gene is induced after BaMV infection with the highest level at 7 dpi, whereas it remains unchanged in non-inoculated plants during the infection period (Fig. 1B–C). These data support the cDNA-AFLP result that BaMV infection accelerates NbSTKL expression, especially at a later point of the infection.

To obtain the full-length cDNA of NbSTKL for a further investigation of the protein function, we used the 5′ and 3′ rapid amplification of cDNA ends (RACE) and an RT-PCR based on the sequences deposited in the NCBI gene bank (EX534117). In total, 1732 nts NbSTKL cDNA are acquired and predicted to encode a 413-amino acid-polypeptide in addition to the 217 and 276 nts of the 5′ and the 3′ UTR, respectively (Fig. 1A). Based on the prediction, the ORF contains a PKc domain with an active site, an ATP binding site, and a substrate binding site (data not shown), and is highly homologous to the serine/threonine protein kinase of *Ricinus communis* (71% identity) and of other plants.

### NbSTKL is Involved in BaMV Accumulation in the *N. benthamiana* Leaves

**Figure 2:**
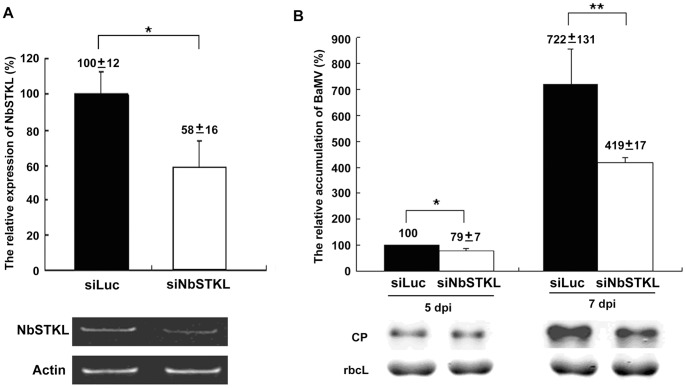
The relative levels of NbSTKL expression and BaMV accumulation in NbSTKL-knockdown *N. benthamiana* plants. **A.** The expression levels of NbSTKL was determined by semi-quantitative RT-PCR (low panel) and actin was used for an internal control. Average from three experiments is shown in the top panel. Actin was used for normalization of each sample. The expression of NbSTKL in Luciferase-knockdown (siLuc) plants was set as 100%. **B.** The accumulation of BaMV coat protein was detected by Western blotting and normalized to the amount of RuBisCO large subunit (rbcL). The samples harvested from 5 dpi control (siLuc) plants were set as 100%. Results were from three independent experiments. The standard errors are shown. Asterisks indicate statistically significant differences between samples indicated (*p<0.05, **p<0.01).

We further investigated whether knockdown of NbSTKL expression influences BaMV accumulation. Taking advantage of the efficient silencing activity of virus-induced gene silencing (VIGS), we used the *Tobacco rattle virus* (TRV) system [33], [34] to convey the partial fragment of NbSTKL derived from cDNA-AFLP (Fig. 1A) to knock down the NbSTKL expression in plants. Ten days after agro-infiltration, the fourth and fifth leaves above the infiltrated leaves were used to inoculate the BaMV viral particles. Silencing efficiency is determined by semi-quantitative RT-PCR using another set of primers beyond the VIGS fragment to avoid the mis-amplification of the infiltrated DNA fragment. The results indicate that approximately 40% of NbSTKL expression is reduced after VIGS (Fig. 2A). Accumulation of the virus is monitored by BaMV coat protein detection at 5 and 7 dpi. Correlated with the silencing efficiency, BaMV accumulation is decreased by 20% and 40%, compared to that of the control plants (Luc) at 5 and 7 dpi, respectively, (Fig. 2B). These results suggest that NbSTKL is positively involved in BaMV accumulation.

Moreover, the growth or development of *N. benthamiana* is not affected after knocking down the NbSTKL gene expression according to the phenotype comparison with non-silenced plants. Therefore, knocking down the expression of NbSTKL may not have significant physiology effect on plants and thus the gene-knocked down effect on BaMV accumulation is further studied.

### NbSTKL has Little Effect on BaMV Replication

**Figure 3:**
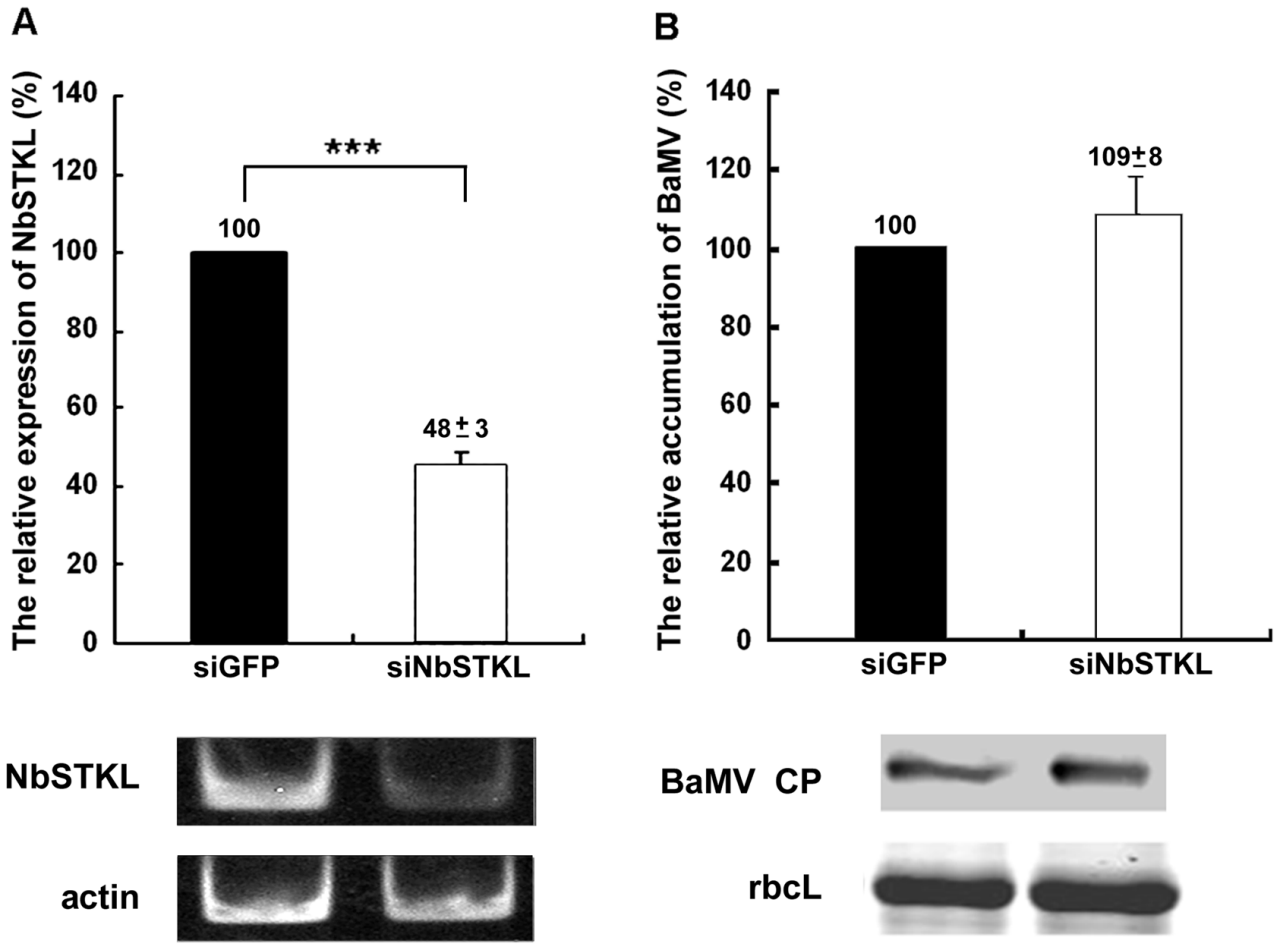
BaMV coat protein accumulation in NbSTKL-knockdown protoplasts. **A.** The knockdown efficiency was measured by semi-quantitative RT-PCR and normalized to the actin. GFP-knockdown (siGFP) was set as 100%. RT-PCR results of NbSTKL expression and the actin internal control are shown under the statistic bar. **B.** BaMV coat protein accumulation level was determined by Western blotting and normalized to that of RuBisCO large subunit (rbcL). The control (siGFP) and NbSTKL-knockdown protoplast (siNbSTKL) were inoculated with BaMV viral RNA. The coat protein accumulation from control protoplasts was set as 100%. Asterisks indicate statistically significant differences between samples indicated (***p<0.001). The Western results of BaMV coat protein accumulation (BaMV CP) and the Coomassie Brilliant Blue staining of RuBisCO large subunit (rbcL) are shown under the statistic bar.

Knocking down NbSTKL expression in *N. benthamiana* plants leads to a reduction of BaMV accumulation, which could be due to a defect in virus replication and cell-to-cell movement. To distinguish the step in the BaMV infection cycle in which NbSTKL is involved, we isolated protoplasts from NbSTKL-knockdown *N. benthamiana*, followed by BaMV inoculation. The expression of NbSTKL was reduced to approximately 50% in the protoplasts, as observed in the inoculated leaves (Fig. 3A). After 24 h of virus inoculation, the coat protein accumulation levels of BaMV show no difference between the knockdown and control protoplasts (Fig. 3B). This finding demonstrates that NbSTKL may not participate in BaMV replication but rather in the cell-to-cell movement of BaMV.

### Cell-to-cell Movement of BaMV is Restricted in the NbSTKL-knockdown Leaves

**Figure 4:**
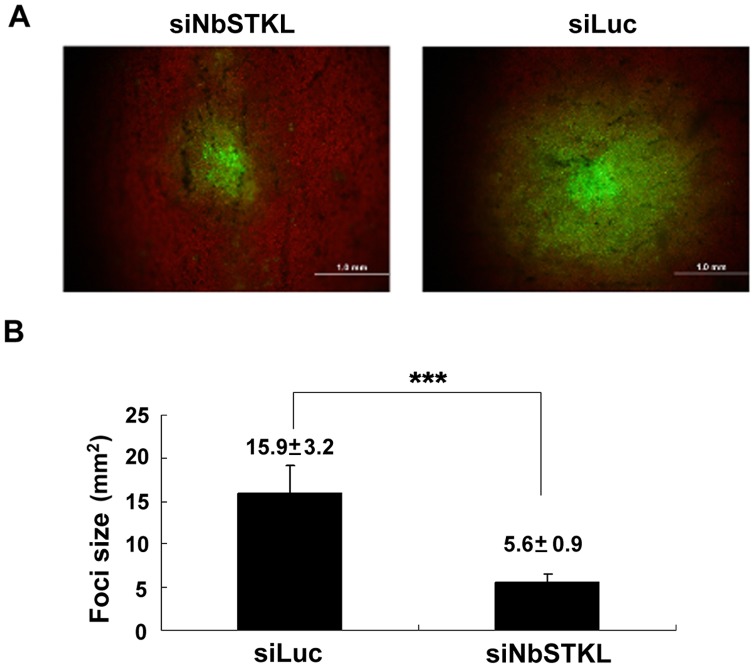
Cell-to-cell movement of BaMV-GFP in the NbSTKL-knockdown *N. benthamiana* leaves. BaMV-GFP plasmids were inoculated onto the NbSTKL- knockdown (siNbSTKL) or control (siLuc) plants. The sizes of GFP foci were measured. **A.** The sizes of GFP foci in the inoculated leaves of NbSTKL-knockdown and control plants. **B.** Statistic analysis of the foci measured from A. Asterisks indicate statistically significant differences between samples indicated (***p<0.001).

To obtain direct evidence of NbSTKL involvement in BaMV cell-to-cell movement, we inoculated the NbSTKL- and Luc-knockdown (control) leaves with GFP-expression BaMV, an infectious clone of BaMV with GFP expression from a duplicated coat protein promoter [26]. Therefore, we can follow the BaMV movement by observing GFP signals. From the sizes of the GFP foci that we measured, BaMV was more restricted in NbSTKL-knockdown leaves than in the control leaves (Fig. 4A). The average size of the foci on the NbSTKL-knockdown leaves was approximately 5.6 mm^2^, whereas that of the foci on the control leaves was 15.9 mm^2^ (Fig. 4B). These data demonstrate that NbSTKL is involved in BaMV cell-to-cell movement and less expression of NbSTKL in plants confines BaMV cell-to-cell movement.

### Active Site Mutation of NbSTKL Affects BaMV Accumulation in Plants

**Figure 5:**
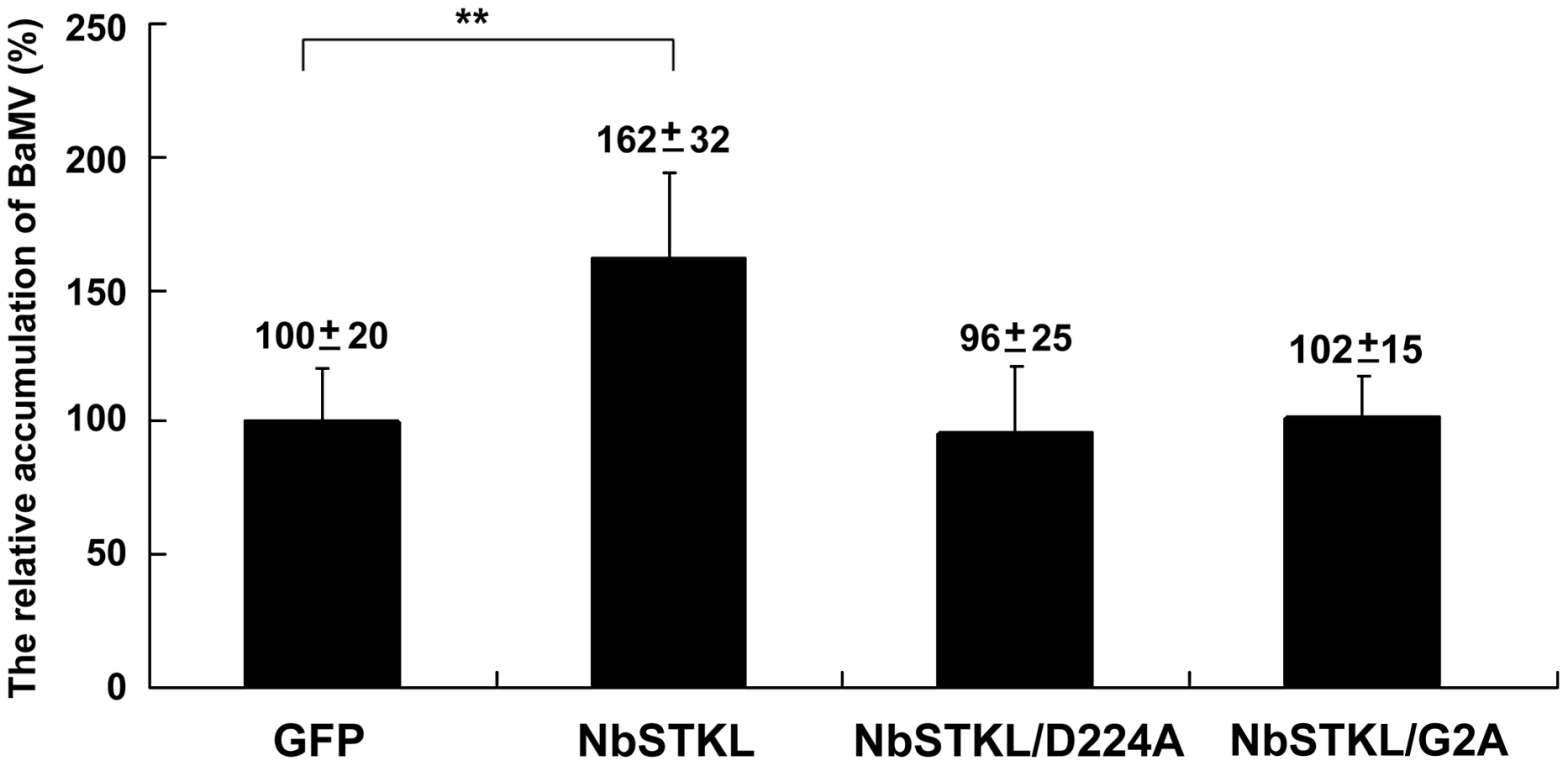
Effect of NbSTKL and its derivatives on BaMV accumulation. BaMV was inoculated onto the *N. benthamiana* leaves transiently expressed NbSTKL and its derivatives NbSTKL/D224A and NbSTKL/G2A. The coat protein accumulation was detected by Western blotting and normalized to the RuBisCO large subunit (rbcL). GFP transient expressed-leaves were also inoculated with same amount of BaMV and set as 100%. Asterisks indicate statistically significant differences between samples indicated (**p<0.01).

To demonstrate that kinase activity is required for BaMV accumulation in plants, we transiently expressed an active site mutant NbSTKL/D224A (amino acid 224 aspartic acid changed to alanine) in *N. benthamiana* leaves followed by BaMV inoculation. Western blotting shows that the expression of wild-type (WT) NbSTKL could enhance the accumulation to 1.6-fold compared to that of the expressed GFP control, whereas the active site mutant (NbSTKL/D224A) in plants did not show any enhancement (Fig. 5). These results imply that the kinase-like activity of NbSTKL is vital for facilitating BaMV accumulation in *N. benthamiana* plants.

### NbSTKL is a Membrane-associated Protein

**Figure 6:**
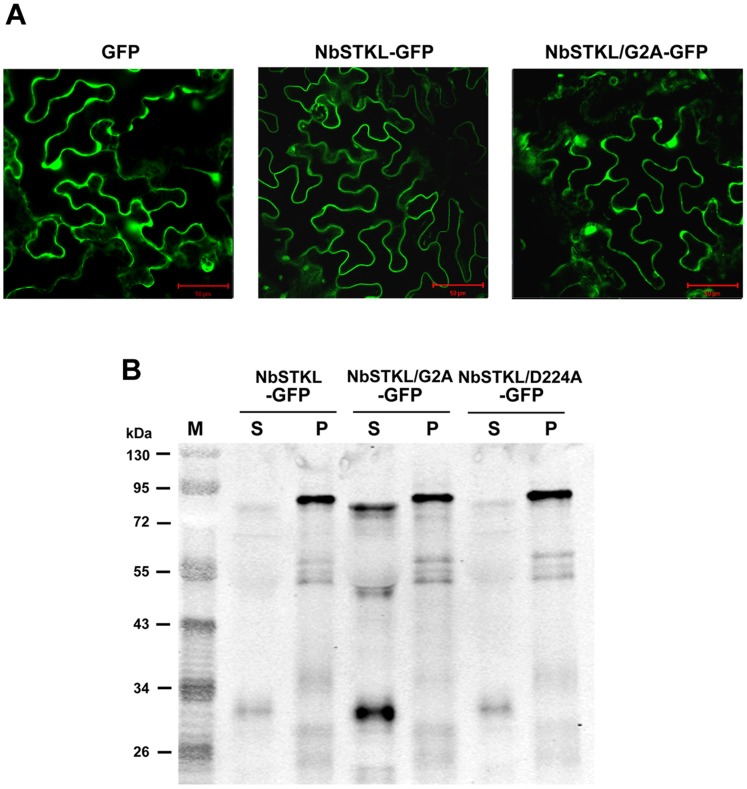
The subcellular localization of NbSTKL. **A.** Subcellular localization of GFP, NbSTKL-GFP and NbSTKL/G2A-GFP was observed by confocal microscopy. Images were obtained by Olympus Fluoview FV1000 confocal microscope with 488 nm excitation. **B.** Western blotting was used to detect the localization of NbSTKL and its derivatives. The GFP fused NbSTKL (NbSTKL-GFP) and its derivatives NbSTKL/G2A-GFP and NbSTKL/D224A-GFP were transiently expressed by agro-infiltration onto *N. benthamiana* leaves. The total proteins were extracted and separated into the soluble fraction (S) and the pellet fraction (P). GFP antibody was used for Western blot assay. The scale bars represent 50 μm.

After discovering that NbSTKL regulates BaMV cell-to-cell movement, we speculated that NbSTKL could be associated with the membrane. To gain insight into the subcellular localization of NbSTKL, GFP was fused to the C-terminus of NbSTKL (NbSTKL-GFP) and transiently expressed by agro-infiltration. Confocal microscopy indicated that NbSTKL-GFP is lying along the edge of the cell more likely to be the membrane associated characteristic in comparison to that of GFP alone (Fig. 6A). Centrifugation to separate the cytoplasm and cell wall (membrane) fractions showed that, unlike free GFP, which appears mostly in the cytoplasm, NbSTKL-GFP is mainly remained in the pellet (Fig. S1). After treating with non-ionic detergent, 2% Triton X-100 or 2% NP40, the majority of NbSTKL-GFP still remains in the pellet (data not shown), a phenomenon also observed in poliovirus capsid VP4 protein [35]. These results indicated that NbSTKL is strongly associated with membrane.

Because NbSTKL is associated with the membrane without obvious hydrophobic domain, we have checked the amino acid sequence and showed a G2/C4 (second glycine and fourth cysteine) motif at the N-terminal region that has been reported to be myristoylated at G2 and palmitoylated at C4 [36]–[38]. Based on this prediction, we created the G2A mutant that the localization image was more similar to that of GFP alone (Fig. 6A) and approximately 50% of NbSTKL/G2A-GFP was solubilized (Fig. 6B). Mutant NbSTKL/D224A-GFP kept the G2/C4 motif remained in a membrane-associated characteristic. The results also indicated that the soluble form of NbSTKL is somehow smaller than that in the membrane-bound form. The difference in size could be due to the uncharacterized modification after anchoring to the membrane [39], [40]. Furthermore, the membrane anchoring of NbSTKL is also playing an essential role in helping the BaMV movement. Transiently expressed NbSTKL/G2A could not assist the accumulation of BaMV as that of wild-type (Fig. 5).

### NbSTKL has Kinase Activity for Autophosphorylation

**Figure 7:**
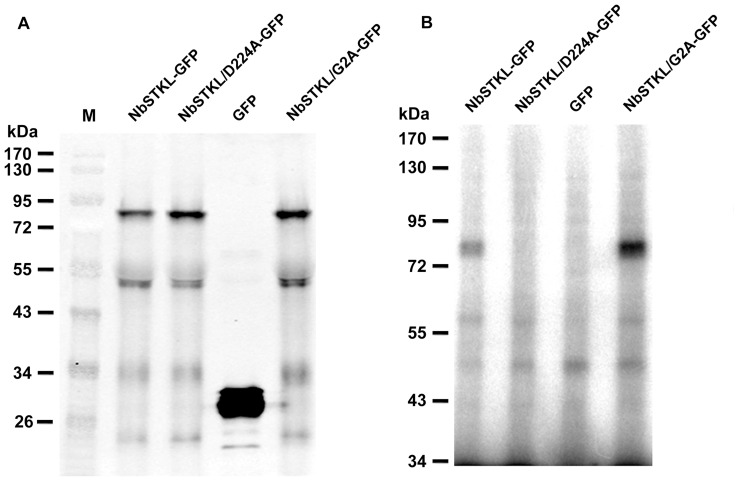
The kinase activity assay of NbSTKL and its derivatives. **A.** The total proteins derived from *N. benthamiana* plants, transiently expressed GFP, NbSTKL or its derivatives as indicated, were subjected to Western blotting analysis with anti-GFP antibody. **B.** The soluble fraction of each protein extract was trapped with anti-GFP magnetic beads and incubated with [γ-^32^P]ATP for autophosphorylation assay.

To investigate whether NbSTKL has kinase activity, NbSTKL-GFP was transient expressed in *N. benthamiana* and tested for autophosphorylation. NbSTKL-GFP was trapped by anti-GFP megnetic beads and subjected for kinasing activity assay. At a size approximately 75 kDa, as the predicted size of NbSTKL-GFP (Fig. 7A; total protein loaded for western blotting with anti-GFP antibody), a clear radioactive band is observed in the NbSTKL-GFP reaction but not in the GFP control nor in the active site mutant (NbSTKL/D224A-GFP) (Fig. 7B). Furthermore, mutant NbSTKL/G2A-GFP showed more soluble form in cells (Fig. 6) had more kinasing activity (Fig. 7B).

### Transient Expression of NbSTKL does not Significantly Affect the Accumulation of *Potato Virus X* and *Cucumber Mosaic Virus*

**Figure 8:**
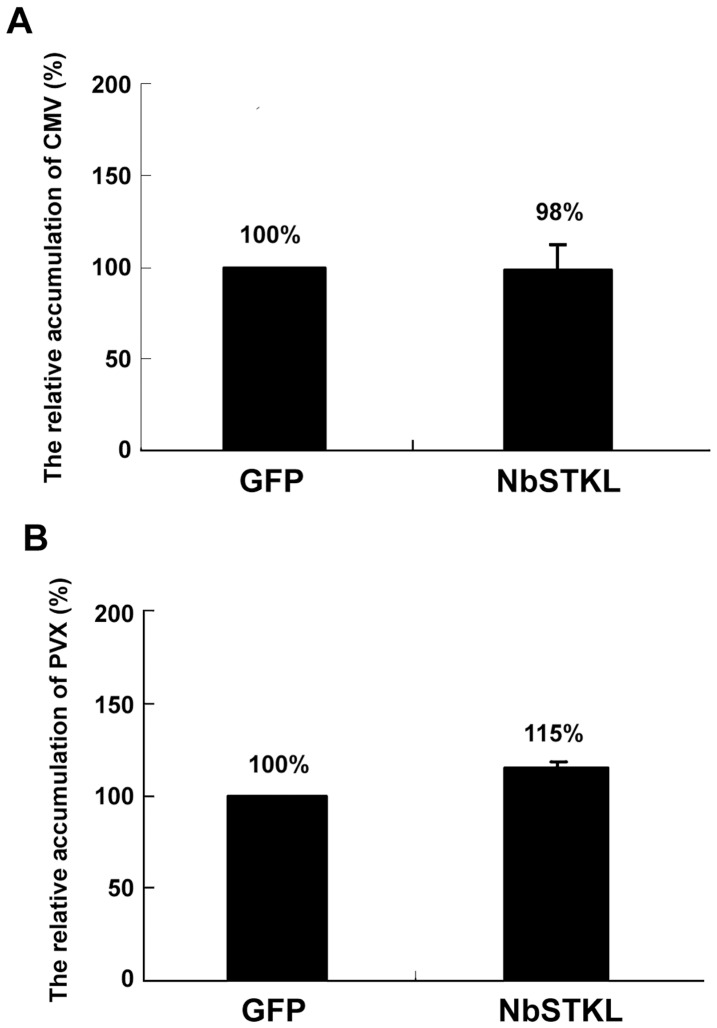
PVX and CMV coat protein accumulation in the NbSTKL transiently expressed-*N. benthamiana*. The control (GFP) and NbSTKL transiently expressed-plants (STK) were inoculated with PVX or CMV viral particles at the transiently expressed leaves. The coat protein accumulation levels were detected by **A.** PVX or **B.** CMV coat protein antibody and normalized to the RuBisCO large subunit. The accumulation levels of viral coat protein in GFP control leaves were set as 100%. All data were the averages of three independent experiments and the standard errors are shown.

Because numerous kinases are involved in viral infection cycles [14], [21], [41], we tested whether the identified NbSTKL also regulates the accumulation of PVX and *Cucumber mosaic virus* (CMV). Unlike the dramatically increased effect of NbSTKL transient expression on BaMV accumulation, neither PVX nor CMV is interfered with after transiently expressing NbSTKL (Fig. 8). Therefore, NbSTKL is a specific kinase-like protein that regulates the cell-to-cell movement of BaMV, but not those of PVX and CMV. Because the alteration of NbSTKL expression could only specifically regulate the movement of BaMV but not PVX, the route in controlling the virus movement through the action of NbSTKL or its orthologs could not generally be applied to other members of Potexvirus.

## Discussion

In this study, we demonstrated that NbSTKL, a putative protein kinase of *N. benthamiana*, is involved in BaMV cell-to-cell movement. First, we confirmed that the expression profile of *NbSTKL* is upregulated at 5 and 7 dpi of BaMV in *N. benthamiana*, as shown in the cDNA-AFLP experiment (Fig. 1). Induction of the host gene expression could be caused by either a defense of pathogen infection, or conversely, the host gene product is required for pathogen infection. Knocking down NbSTKL expression resulted in a reduced accumulation of BaMV (Fig. 2), suggesting that NbSTKL was involved in BaMV infection rather than in host defense.

We demonstrated that knocking down NbSTKL expression does not have a significant effect on virus replication (Fig. 3). However, when BaMV-GFP is used to monitor the spread of BaMV in NbSTKL-knockdown leaves, the GFP foci are smaller than of those in the control leaves (Fig. 4). These data indicate that the reduction of BaMV coat protein accumulation (Fig. 2) is due to the restriction of viral spreading from cell to cell rather than virus replication. Furthermore, by mutating the putative active site of NbSTKL, we also show that the influence on BaMV cell-to-cell movement is highly correlated to its kinase-like activity (Fig. 5), because the transient expression of NbSTKL-GFP significantly increases BaMV accumulation but not that of active site mutant NbSTKL/D224A-GFP, indicates that the putative activity site of NbSTKL is important for BaMV accumulation. However, the putative active site mutant NbSTKL/D224A may not be a dominant negative mutant; therefore, little effect on BaMV accumulation was observed (Fig. 5).The membrane-association characteristic of NbSTKL (Fig. 6) also makes it a candidate in controlling the viruses from spreading to neighboring cells. However, we did not rule out that NbSTKL is further targeted to the cell wall since the non-ionic detergents were not able to solubilize NbSTK from pellet. Because the transient expression of NbSTKL does not significantly raise the accumulations of PVX and CMV compared to that of BaMV (Fig. 7), NbSTKL may not be a general host protein, which is required for the infection of other viruses.

Virus MP or coat proteins involved in cell-to-cell movement are often the targets of cellular kinases [9], [14]. Phosphorylation of the MP of several plant viruses has been shown to regulate virus cell-to-cell movement [13], [15]. In a previous study on PVX, a kinase of *N. tabacum* with casein kinase 2-like activity was shown to phosphorylate the movement protein TGBp1 *in vitro* [21]. An attempt to investigate the *in vivo* phosphorylation (a metabolic labeling with ^33^P-orthophosphate) of the BaMV TGBp1 movement protein P28 was unsuccessful in the protoplast assay. A possibility is that P28 phosphorylation is not required during viral replication. However, we did not rule out the possibility that in the protoplasts, the expression level of P28 was too low and phosphorylation was under a detectable limit. Considering that P28 is localized mainly in the cell-wall fraction [28] and that several amino acids of P28 have high phosphorylation potential based on a prediction by the NetPhos 2.0 Server, the phosphorylation status of P28 on the cell-wall is compelling and necessary to determine the mechanism of how NbSTKL regulates BaMV cell-to-cell movement. However, at this stage, we do not know if the phosphorylation of P28 is through NbSTKL or other protein kinases.

The cell-to-cell movement of BaMV is thought to occur by transporting the viral RNA complex through the plasmodesmata, the same as in PVX and TMV. Therefore, we speculate that certain kinases should be associated with the plasmodesmata that regulate the passage of BaMV. However, the NbSTKL we identified in this study has little sequence similarity with the tobacco CK2 α subunit identified in the PVX study [21] or to the plasmodesmal-associated protein kinase of Arabidopsis [14], or to tobacco CK2-like kinase [41], which have been shown to phosphorylate TMV MP.

In conclusion, we identified a membrane-associated serine/threonine kinase-like protein from *N. benthamiana* involved in BaMV cell-to-cell movement. In brief, this kinase confines the spread of BaMV but not that of PVX or CMV.

## Supporting Information

Figure S1 **The fractionation of the transiently expressed NbSTKL-GFP in plants.** Western blotting was used to detect the localization of NbSTKL. The GFP fused NbSTKL (NbSTKL-GFP) and the GFP control were transiently expressed by agro-infiltration onto *N. benthamiana* leaves. The total proteins were extracted and separated into the cytoplasm (supernatant) and the membrane fractions. GFP antibody was used for Western blot assay.
(TIF)
