# Supplementary figures and images for: Ser/Thr Kinase-Like Protein of Nicotiana benthamiana Is Involved in the Cell-to-Cell Movement of Bamboo mosaic virus
Source: PLoS One. 2013 Apr 30;8(4):e62907. doi: 10.1371/journal.pone.0062907 (PMC3639906; doi:10.1371/journal.pone.0062907)

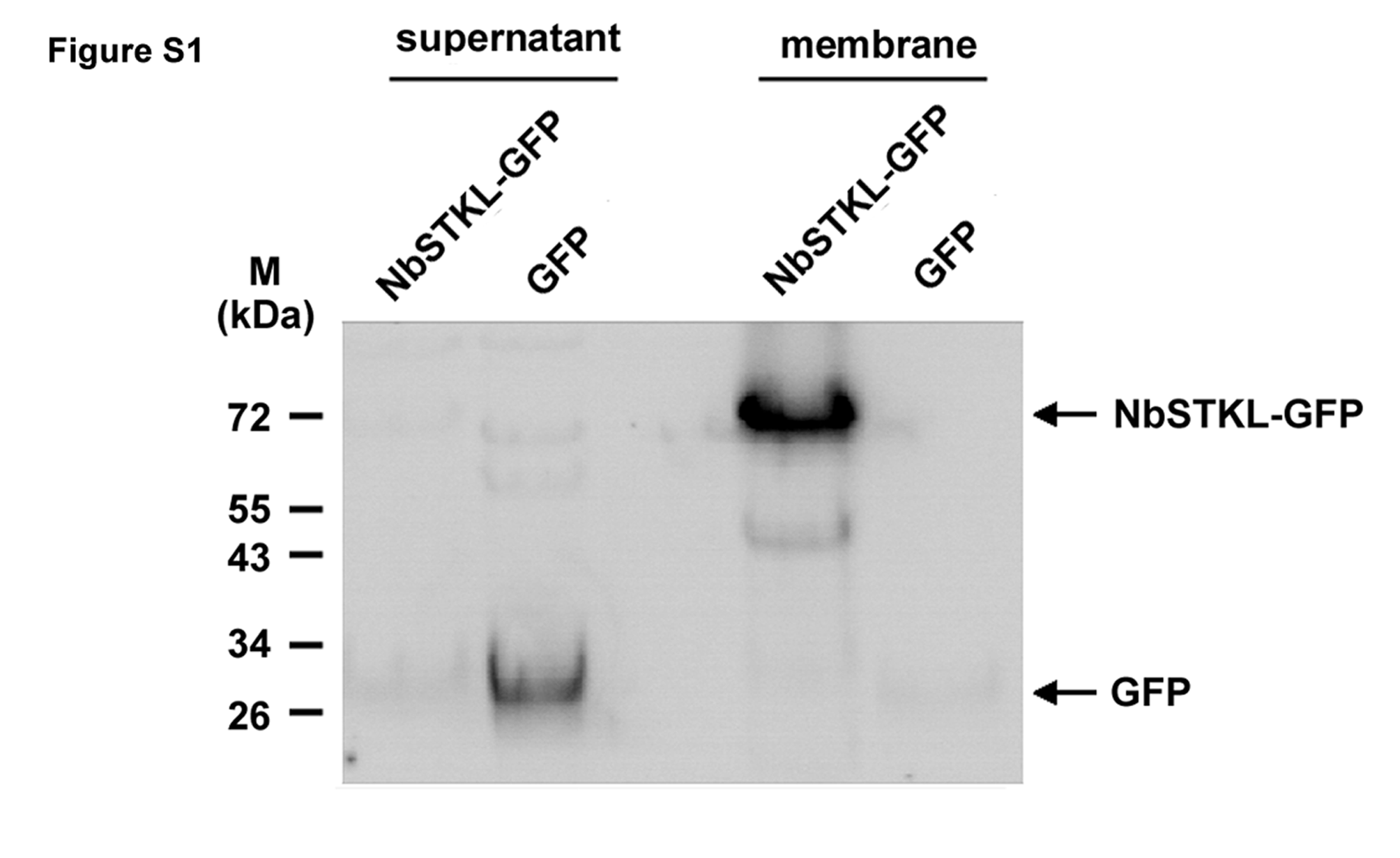

Supplement: Figure S1 — The fractionation of the transiently expressed NbSTKL-GFP in plants. Western blotting was used to detect the localization of NbSTKL. The GFP fused NbSTKL (NbSTKL-GFP) and the GFP control were transiently expressed by agro-infiltration onto N. benthamiana leaves. The total proteins were extracted and separated into the cytoplasm (supernatant) and the membrane fractions. GFP antibody was used for Western blot assay. (TIF) [file pone.0062907.s001.tif]
